# Real-world effectiveness of high-efficacy therapies in multiple sclerosis: a propensity score-matched cohort study

**DOI:** 10.3389/fneur.2026.1773074

**Published:** 2026-03-13

**Authors:** Jasmin Wich, Tatjana Beppler, Michelle Dreiling, André Scherag, Matthias Schwab, Florian Rakers

**Affiliations:** 1 Department of Neurology, Jena University Hospital, Jena, Germany; 2 Institute of Medical Statistics, Computer and Data Sciences, Jena University Hospital, Jena, Germany

**Keywords:** disease-modifying therapies, high-efficacy treatments, multiple sclerosis, propensity score matching, real-world evidence

## Abstract

**Background:**

Effectiveness classification of disease-modifying therapies (DMTs) in MS relies either on head-to-head trials or indirect comparisons of annualized relapse rates (ARR) across studies. Validating efficacy estimates in real-world settings is crucial, particularly as the discussion on the broader use of high-efficacy (HE) DMTs including antibodies, S1P receptor modulators, and cladribine tablets, continues to evolve. Because treatment decisions in clinical practice are individualized and no single DMT is universally preferred, effectiveness assessments are appropriately conducted at the group level.

**Objective:**

To evaluate the real-world effectiveness of HE vs. low-efficacy (LE) DMTs at the group level.

**Methods:**

A retrospective 1:1 propensity score-matched cohort study was conducted using routine data from MS patients initiating HE or LE DMTs at a German tertiary MS center between 2007 and 2023. Primary outcome was ARR; secondary endpoints were cumulative hazard of relapse, MRI activity, loss of NEDA-3, 3-month confirmed disability progression (CDP), and treatment discontinuation.

**Results:**

After matching, 266 datasets were analyzed with a median follow-up of 24 months in the LE DMT group and 43 months in the HE DMT group. ARR was 64% lower with HE DMTs (0.13 vs. 0.37; rate ratio 0.36, 95% –CI: 0.23–0.53; *p* < 0.001). Relapse hazard, MRI activity, and NEDA-3 loss were 53–63% lower (hazard ratios 0.41, 0.37, and 0.47; all *p* < 0.001). CDP did not differ between groups. Hazard of treatment discontinuation was 67–87% lower with HE DMTs (hazard ratios 0.13–0.33; all *p* < 0.001). No discontinuation due to recurrent infections occurred.

**Conclusion:**

HE DMTs resulted in significantly lower clinical and radiological disease activity than LE DMTs in real-world conditions while showing greater treatment persistence. These findings support the broad use of HE DMTs, while LE DMTs may be reserved for specific indications.

## 1 Introduction

Over the past two decades, the therapeutic landscape for multiple sclerosis (MS) has evolved significantly. Today, nine distinct classes of disease-modifying therapies (DMTs) with varying mechanisms of action are available for relapsing-remitting MS (RRMS), commonly classified into high (HE) or low efficacy (LE) therapies (1–3).

DMT efficacy classification primarily relies on two approaches, both with inherent limitations. The first, considered the highest level of evidence, is based on head-to-head randomized controlled trials (RCTs) demonstrating the superiority of one DMT over another, typically using annualized relapse rate (ARR) as the primary outcome. However, some HE DMTs, such as cladribine and natalizumab, have only been tested against placebo (4, 5), while no HE DMT trial has included glatiramer acetate or fumarates as active comparators, leaving gaps in direct comparative evidence (6). In the absence of head-to-head comparisons, the second approach estimates DMT efficacy by comparing ARR reductions across different RCTs, as implemented in several MS guidelines (7–9). However, this approach is controversial, as differences in study design, patient populations, and follow-up time complicate cross-trial comparisons and also challenges the validity of network meta-analyses (10). Moreover, RCTs are conducted in highly controlled settings that do not reflect real-world conditions. Given these limitations, validating current efficacy estimates in real-world studies remains essential, particularly as the discussion on a broader use of HE DMTs, even early in the disease course, continues to evolve (11–15).

To date, a number of observational studies have reproduced the results obtained by RCTs by comparing HE vs. LE DMTs in real-word cohorts. Most of these studies compared individual HE DMTs with individual LE DMTs and demonstrated superior reductions in ARR with HE therapies (16–18). However, treatment selection in clinical practice is influenced not only by efficacy but also by medical considerations and patient preferences, and no single DMT is universally favored. Consequently, evaluating treatment effectiveness at the level of efficacy groups rather than individual agents represents a clinically meaningful approach. This concept is reflected in current MS guidelines and expert statements, which predominantly distinguish therapies according to efficacy categories rather than individual compounds (11–13). Two large real-world studies have previously demonstrated superior relapse reduction with HE compared to LE DMTs at the group level (14, 15). However, these studies were restricted to treatment-naïve patients and therefore do not fully reflect real-world treatment patterns, where therapy switches are common.

The present study addresses this gap by comparing real-world effectiveness of HE vs. LE DMTs at the group level in reducing clinical and radiological disease activity in a cohort including both treatment-naïve patients and patients switching from prior therapies. We applied propensity score (PS) matching to adjust for baseline differences in patient characteristics and minimize treatment selection bias. Additionally, we evaluated treatment discontinuation as a surrogate safety marker and describe the associated reasons for treatment discontinuation.

## 2 Materials and methods

### 2.1 Study population

This retrospective, observational cohort study compared the real-world effectiveness of HE vs. LE DMTs in patients with RRMS. In accordance with a common classification (3, 6, 11), HE DMTs comprised fingolimod, ozanimod, cladribine, natalizumab, alemtuzumab, ocrelizumab, and ofatumumab, while LE DMTs included beta-interferons, glatiramer acetate, dimethyl/diroximel fumarate, and teriflunomide.

**Figure 1:**
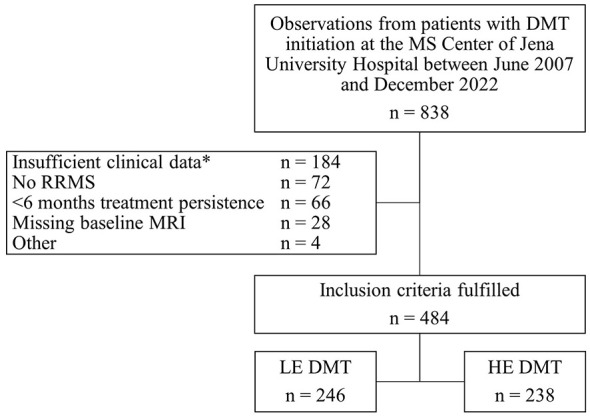
Flowchart of the study recruitment process. DMT: disease-modifying therapy; HE, high-efficacy; LE, low-efficacy; MRI, magnetic resonance imaging; RRMS, relapsing-remitting multiple sclerosis. *e.g. missing follow-up data, missing EDSS baseline values.

The study recruitment process is shown in Figure 1. Patients were included if they initiated DMT between June 2007 and December 2022 at the tertiary MS center of Jena University Hospital/Germany as part of routine care. Demographic and clinical data, including MRI results, were retrospectively obtained from clinical databases via manual extraction and verified by chart review. Data sources included electronic medical records and physician's letters, with August 2023 as the last collection date. The local institutional review board approved the study (No. 3639-BOD). Data were anonymized before analysis; written informed consent was not required.

Inclusion criteria were: (1) RRMS diagnosis per applicable McDonald criteria at diagnosis; (2) initiation of a study DMT at the Jena MS center as part of routine care; (3) ≥6 months of treatment to allow observation of therapeutic effects (19); (4) a minimum dataset with follow-up data from ≥1 year before and ≥6 months after DMT initiation (including sex, age, disease course, time since first MS symptoms, relapse/progression dates, and DMT history); (5) ≥3 disability assessments, with ≥1 expanded disability status scale (EDSS) score within six months before and one month after DMT start (baseline EDSS) and ≥2 EDDS scores during follow-up, recorded ≥3 months apart, with ≥1 assessed during treatment; (6) a cerebral MRI within 12 months before and one month after DMT initiation (baseline MRI).

### 2.2 Procedures

The MS center's clinical databases contain data from routine procedures that follow internal standards aligned with national MS guideline recommendations (9). DMT was administered by board-certified neurologists, following the respective summary of product characteristics. Clinical visits with systematic recording of disease-related data were scheduled every 3–6 months. EDSS scores were derived from clinical examinations, typically by the same neurologist. MRI scans were generally performed annually (cerebral in all cases, spinal if indicated) and evaluated by certified neuroradiologists. Contrast agents were routinely used unless contraindicated.

### 2.3 Outcome measures

The primary endpoint of the study was the ARR. Secondary endpoints included the cumulative hazard of relapse, 3-month confirmed disability progression (CDP), MRI activity, loss of ‘no evidence of disease activity' status (NEDA-3), and treatment discontinuation.

The observation unit was the MS patient and their respective DMT. Cohort entry was defined as the date of first dispensing of the respective DMT under observation and thus did not necessarily represent the patient's first-ever DMT. Follow-up ended at DMT discontinuation/switch, last recorded visit (loss to follow-up/administrative censoring), or, for time-to-event endpoints, the event itself. A treatment switch initiated a new observation unit. Consequently, some patients constitute multiple observations, which was accounted for in sensitivity analysis. Treatment discontinuation was defined as a documented clinical decision to stop or switch the initiated DMT and was not inferred from dosing intervals or prescription supply.

ARR was defined as number of relapses divided by years observed for each treatment group (20). A relapse was defined as new or exacerbated MS symptoms lasting ≥24 h, occurring >30 days after the onset of a prior relapse, and not accompanied by fever or infection (21). CDP was identified as an EDSS increase of 1 point (1.5 points if baseline *EDSS* = 0; 0.5 points if baseline EDSS >5.5), and confirmed ≥3 months later (22). EDSS scores ≤ 30 days post-relapse were excluded. MRI activity included new/enlarging T2 hyperintense lesions or contrast-enhancing lesions on cerebral/spinal MRI. T2 hyperintense lesions were manually counted and categorized as 1–2, 3–8, or >9. NEDA-3 required the absence of relapses, CDP, or MRI activity; loss of NEDA-3 was defined as the occurrence of any of these events (23).

### 2.4 Statistical analysis

We utilized PS matching to address non-randomized treatment allocation. PS was estimated using multivariable logistic regression, with baseline covariates as independent variables and the allocated DMT group as a binary dependent variable. Covariates were selected based on domain knowledge to include factors likely influencing the outcome (24). The model included sex, age at baseline, time since first MS symptoms, baseline EDSS, prior-year relapses (count) and CDP (yes/no), number of MRI lesions (categorized), contrast-enhancing lesions (present or absent/unknown), number of prior DMTs, and most effective prior DMT (none/LE/HE). Cases with missing values in baseline covariates were excluded to ensure robust PS estimation (Figure 1). Baseline MRIs without contrast agents were classified as contrast-negative, affecting < 7% of cases in both groups.

Matching was performed using 1:1 nearest neighbor matching without replacement within a caliper of 0.2 standard deviations of the propensity score (25, 26), implemented using the MatchIt R package (27). Matching was conducted on the propensity score rather than its logit, thus achieving better covariate balance. Balance between matched groups was assessed using absolute standardized mean differences (SMDs), with SMD ≤ 0.1 indicating excellent balance and higher values being tolerated for less prognostic covariates (28). Matching effectiveness and covariate balance were further evaluated using density plots of PS covariates and z-differences (29).

Cohort characteristics were summarized as median with interquartile range (IQR) for continuous variables and counts (%) for categorical variables. ARRs and 95% confidence intervals (CI) were estimated using a Quasi-Poisson model, accounting for overdispersion in count data (30); robustness of the results was further evaluated in a sensitivity analysis using a negative binomial model. ARR comparison was performed using the rate ratio, where significance was tested using a Wald test. Time-to-event analyses were performed using the Kaplan-Meier estimator, and log-rank tests were used for survival curve comparisons. Survival curves were truncated at 5 years, as < 10%−20% cases remained at risk beyond this time (31). Hazard ratios and 95%-CI for relapse, CDP, MRI activity, NEDA-3 loss, and treatment discontinuation were estimated using Cox proportional hazards models, with significance tested using likelihood-ratio tests. The proportional hazards assumption was evaluated using Schoenfeld residual test and graphical methods (e.g., log-minus-log plots). If violated, a step function was used to model time-dependent coefficients (32). Consequently, hazard ratios for treatment discontinuation were calculated for two intervals: 6–12 months and >1 year post-DMT initiation. DMT persistence and reasons for discontinuation were assessed descriptively. For MRI and NEDA-3 analyses, follow-up time was truncated at the last visit involving an MRI and cases without ≥1 follow-up MRI were excluded (5 observations [4%] on HE DMT, 10 observations [8%] on LE DMT). Baseline comparisons confirmed this exclusion had no impact on covariate balance. An exploratory *post-hoc* analysis compared DMT effectiveness within the HE group (Cladribine/S1P receptor modulators *vs*. antibody-based DMTs) following the methodology of the main analysis (i.e., renewed PS matching).

Sensitivity analyses assessed robustness against potential biases. To account for group differences in follow-up time, pairwise censoring was applied, with follow-up right-censored to the shorter observation time within each propensity score pair. The impact of MRI frequency on MRI activity and visit frequency on CDP was addressed using an adjusted Cox model. Further analyses examined the influence of structural dependencies, including potential correlations in outcomes between observations within matched pairs and between different treatment observations from the same patient. To this end, methods allowing for cluster-robust standard error estimation were used (26). For the ARR, a generalized linear mixed Poisson model was used that included matching stratum and patient ID as random effects. For secondary endpoints, the Cox model was adapted by incorporating them as stratum or cluster variables. The impact of unmeasured confounders on the primary endpoint was evaluated using the *sensemakr* R package (33). Statistical tests were performed at a significance level of 0.05 without correction for multiple comparisons due to the exploratory design (34). Analyses were conducted in R (V.4.4.2).

## 3 Results

### 3.1 Study population

Table 1 presents baseline cohort characteristics and covariate balance diagnostics (SMD) before and after matching. 246 observations from patients receiving LE DMTs and 238 observations from patients receiving HE DMTs met the inclusion criteria. Median follow up time was 31 months for the LE DMT group and 43 months for the HE DMT group (Table 1).

**Table 1:** Cohort characteristics.

**Characteristic**	**Unmatched cohort (n** = **484)**			**PS matched cohort (n** = **266)**		
	**LE DMT (n** = **246)**	**HE DMT (n** = **238)**	**SMD**	**LE DMT (n** = **133)**	**HE DMT (n** = **133)**	**SMD**
**Age** (years), median (IQR)	36 (29–46)	35 (29–43)	0.02	34 (27–44)	36 (29–43)	0.06
**Female sex**, n (%)	178 (72%)	173 (73%)	0.01	100 (75%)	96 (72%)	0.07
**Time since symptom onset** (years), median (IQR)	3.4 (0.6–7.9)	7.0 (2.1–11.6)	0.38	4.6 (1.3–8.8)	4.6 (1.1–9.3)	0.06
Time since MS diagnosis (years), median (IQR)	0.8 (0.1–6.1)	5.3 (1.3–9.8)	0.47	3.0 (0.3–7.6)	2.6 (0.4–7.4)	0.04
**EDSS score**, median (IQR)	1.5 (1.0–2.0)	2.0 (1.5–3.5)	0.63	2.0 (1.0–2.5)	2.0 (1.0–2.5)	0.10
**Relapses last year**, median n (IQR)	1 (0–1)	1 (1–2)	0.36	1 (0–1)	1 (1–1)	0.12
**MRI T2 lesions**, n (%)			0.46			0.07
1–2	15 (6.1%)	2 (0.8%)		1 (0.8%)	2 (1.5%)	
3–8	65 (26%)	33 (14%)		24 (18%)	25 (19%)	
≥9	166 (67%)	203 (85%)		108 (81%)	106 (80%)	
**Contrast enhancing MRI lesions**, n (%)	103 (42%)	108 (45%)	0.07	65 (49%)	62 (47%)	0.05
**CDP last year**, n (%)	50 (20%)	60 (25%)	0.12	27 (20%)	31 (23%)	0.07
**Previous DMT**, median n (IQR)	1 (0–1)	2 (1–2)	0.88	1 (0–2)	1 (0–2)	0.10
**Most effective previous DMT**, n (%)			0.96			0.19
None	123 (50%)	42 (18%)		43 (32%)	42 (32%)	
LE DMT	105 (43%)	103 (43%)		73 (55%)	65 (49%)	
HE DMT	18 (7.3%)	93 (39%)		17 (13%)	26 (20%)	
Follow-up time (months), median (IQR)	31 (14–56)	43 (22–68)	0.33	24 (12–49)	43 (22–70)	0.59
Visit intervals (months), median (IQR)	5 (3–8)	3 (3–5)	0.63	5 (3–7)	3 (3–5)	0.53
MRI intervals (months), median (IQR)	12 (8–15)	12 (10–13)	0.00	11 (7–15)	12 (11–13)	0.15
**Actual DMT, n (%)**						
Interferons	63 (26%)	–	–	34 (26%)	–	–
Glatirameracetat	31 (13%)	–	–	14 (11%)	–	–
Teriflunomide	47 (19%)	–	–	28 (21%)	–	–
Fumarate	105 (43%)	–	–	57 (43%)	–	–
Fingolimod	–	47 (20%)	–	–	25 (19%)	***–***
Cladribine	–	35 (15%)	–	–	21 (16%)	***–***
Ozanimod	–	4 (1.7%)	–	–	4 (3.0%)	***–***
Natalizumab	–	62 (26%)	–	–	33 (25%)	***–***
Alemtuzumab	–	26 (11%)	–	–	15 (11%)	***–***
Ocrelizumab	–	48 (20%)	–	–	23 (17%)	***–***
Ofatumumab	–	16 (6.7%)	–	–	12 (9.0%)	***–***

EDSS, expanded disability status scale; CDP, 3-month confirmed disability progression; DMT, disease-modifying therapy; LE, low-efficacy; HE, high-efficacy; MRI, magnetic resonance imaging; n, number of observations; PS, propensity score; IQR, interquartile range; SMD, absolute standardized mean difference. Covariates used for PS estimation are shown in bold. An SMD of ≤ 0.1 defines adequate balance between the treatment groups.

**Figure 2:**
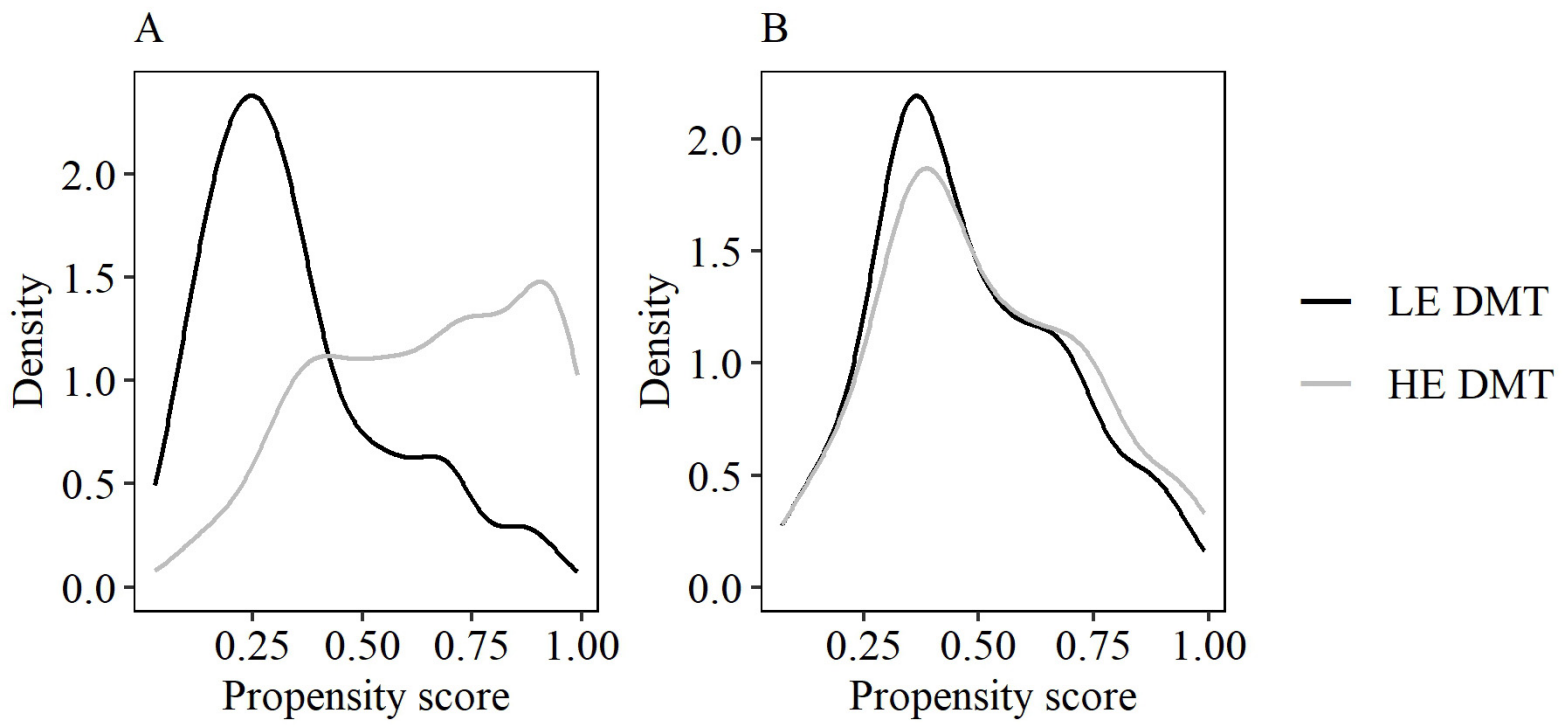
Propensity score overlap in LE and HE DMT cohort **(A)** before and **(B)** after matching. DMT, disease-modifying therapy; LE, low-efficacy; HE, high-efficacy.

**Figure 3:**
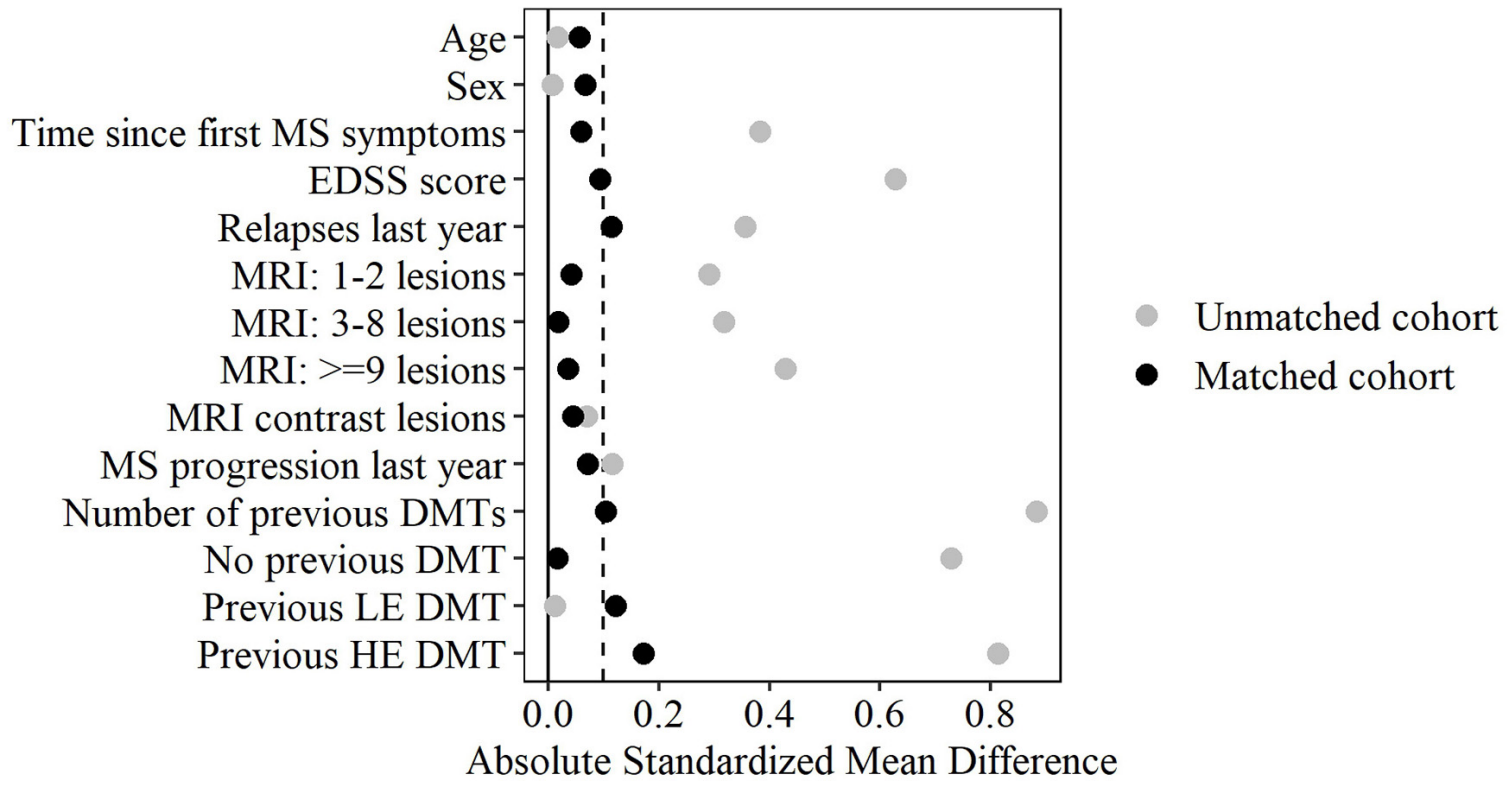
Love plot for covariate balance before and after propensity score matching. CDP: 3-month confirmed disability progression; DMT: disease-modifying therapy; EDSS, expanded disability status scale; HE, high-efficacy; LE: low-efficacy; MRI, cerebral magnetic resonance imaging. Absolute Standardized mean differences are shown individually for multifactorial variables. The vertical dashed line marks the 0.1 threshold for optimal balance.

Most baseline characteristics were imbalanced before PS matching (SMD >0.1, Table 1). HE DMT patients had longer disease duration, greater disability, more relapses and CDP in the previous year, higher MRI lesion burden, more prior DMTs, and a higher proportion of prior HE DMT use. 1:1 PS matching yielded well-balanced cohorts with 133 observations per group and adequate PS overlap (Figure 2). Median follow-up time after propensity score matching was 24 months for the LE DMT group and 43 months for the HE DMT group (Table 1). The love plot (Figure 3) illustrates SMD reductions post-matching. Most SMDs fell below 0.1, indicating excellent balance (Table 1, Figure 3). Minor residual differences remained, with HE DMT patients more likely to have prior HE DMTs and a higher relapse frequency in the preceding year (Table 1, Figure 3).

### 3.2 Primary outcome

**Figure 4:**
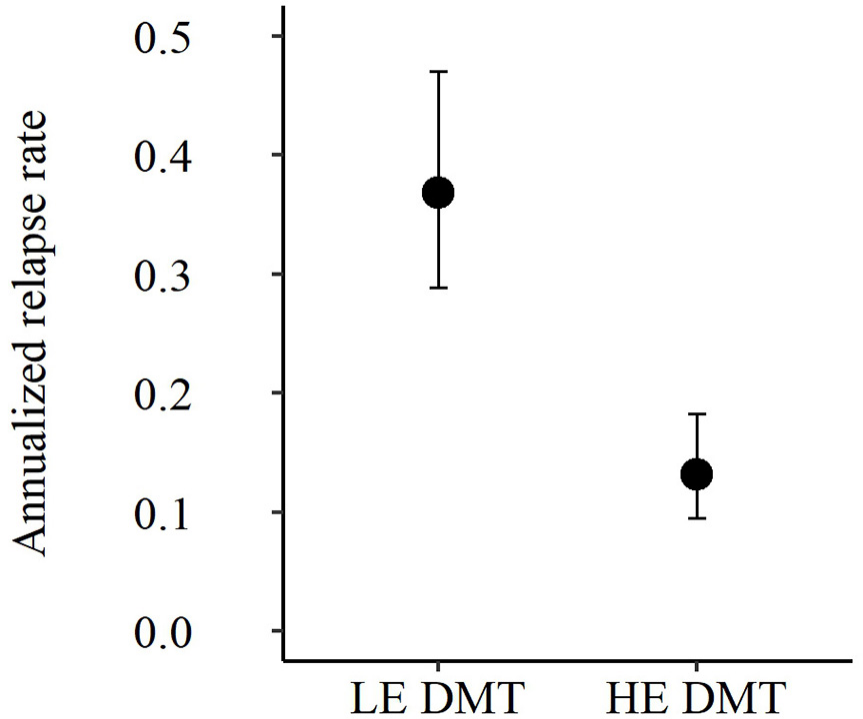
Annualized relapse rates of the PS-matched cohort. DMT: disease-modifying therapy; HE: high-efficacy; LE: low-efficacy; PS: propensity score. Annualized relapse rate estimates with 95% confidence intervals from Quasi-Poisson regression. Estimates were consistent with the crude ARR calculation.

Figure 4 visualizes the treatment group-specific ARRs. The ARR was 64% lower in patients receiving HE DMTs (0.13, 95%-CI 0.09–0.18) compared to those receiving LE DMTs (0.37, 95%-CI 0.29–0.47), corresponding to a rate ratio of 0.36 (95%-CI 0.23–0.53, *p* < 0.001).

### 3.3 Secondary outcomes

**Figure 5:**
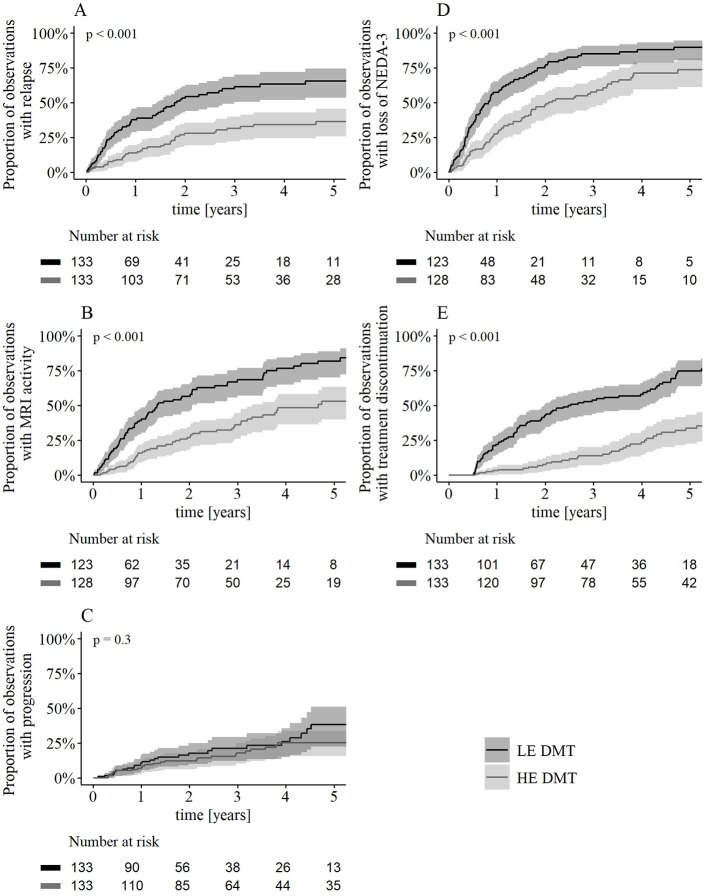
Kaplan–Meier estimates of secondary outcomes. Kaplan–Meier estimates and log-transformed pointwise 95% confidence intervals showing the proportion of observations experiencing **(A)** relapse, **(B)** MRI activity, **(C)** CDP, **(D)** loss of NEDA-3, **(E)** discontinuation over time. *P*-values result from log-rank test. Observations without follow-up MRI were excluded for MRI-based endpoints (**B**, **D**; LE DMT *n* = 10, HE DMT *n* = 5). Patients who discontinued treatment within the first 6 months were excluded by design, as reflected in the Kaplan–Meier plot by a flat line during this period **(E)**. DMT, disease-modifying therapy; LE, low-efficacy; HE, high-efficacy; MRI, magnetic resonance imaging; CDP, 3-month confirmed disability progression; NEDA, no evidence of disease activity.

The cumulative hazard of relapse was 59% lower in patients receiving HE DMTs compared to those receiving LE DMTs (HR 0.41, 95%-CI 0.28–0.60, *p* < 0.001, Table 2, Figure 5A). Similarly, the cumulative hazard of MRI activity was 63% lower in the HE DMT group (HR 0.37, 95%-CI 0.26–0.53, *p* < 0.001, Table 2, Figure 5B) while the hazard of CDP did not differ significantly between groups (Table 2, Figure 5C). HE DMTs resulted in a 53% lower hazard of loss of NEDA-3 compared to LE DMTs (HR 0.47, 95%-CI 0.35–0.64, *p* < 0.001, Table 2, Figure 5D). The cumulative hazard of treatment discontinuation was 87% lower in the HE DMT group during the first year (HR 0.13, 95%-CI 0.04–0.36, *p* < 0.001, Table 2, Figure 5E) and 67% lower from one year onward (HR 0.33, 95%-CI 0.21, 0.50, *p* < 0.001, Table 2, Figure 5E) compared to the LE DMT group. Reasons for discontinuation (Table 3) differed: in the LE DMT group, relapses were the primary cause (45%), while in the HE DMT group, the increased risk of progressive multifocal leukoencephalopathy (PML) during natalizumab treatment was most common (33%).

**Table 2:** Cox proportional hazard models for PS-matched patients with HE and LE DMT.

**Endpoint**	**Hazard ratio**	**95 %-CI**	***p*-value**
Relapse	0.41	0.28–0.60	< 0.001
MRI activity	0.37	0.26–0.53	< 0.001
CDP	0.76	0.45–1.30	0.3
Loss of NEDA-3	0.47	0.35–0.64	< 0.001
Treatment discontinuation			
0.5–1 year	0.13	0.04–0.36	< 0.001
after 1 year	0.33	0.21–0.50	< 0.001

CDP, 3-month confirmed disability progression; CI, confidence interval; DMT, disease-modifying therapy; HE, high-efficacy; LE, low-efficacy; MRI, magnetic resonance imaging; NEDA, no evidence of disease activity. P-values result from the likelihood-ratio test. Hazard ratio < 1 favors HE DMT.

**Table 3:** Descriptive summary of persistence on LE and HE DMT and reasons for discontinuation in the PS matched cohort.

**Investigation**	**LE DMT (*n* = 133)**	**HE DMT (*n* = 133)**
Patients discontinuing therapy (1), *n*	89	39
**Reasons for discontinuation**, ***n*** **(%)**		
Relapse	40 (45%)	6 (15%)
MRI activity	12 (13%)	1 (3%)
Progression	2 (2%)	5 (13%)
Intolerance	19 (21%)	2 (5%)
Abnormal laboratory values	2 (2%)	3 (8%)
Frequent infections	0 (0%)	0 (0%)
Increased PML risk (natalizumab)	0 (0%)	13 (33%)
Cancer	0 (0%)	0 (0%)
Pregnancy/wish for child	8 (9%)	5 (13%)
Patient wish	6 (7%)	4 (10%)

DMT, disease-modifying therapy; HE, high-efficacy; LE, low-efficacy; n, number of observations; PML, progressive multifocal leukoencephalopathy; PS, propensity score. (1) After a minimum therapy of 6 months. Progression either refers to confirmed disability progression or to conversion to a secondary progressive MS disease course.

### 3.4 Sensitivity analyses

All sensitivity analyses confirmed the robustness of the primary findings, with consistent effect directions, preserved significance, and only minor numerical variations. Analysis for unknown confounders yielded a robustness value of 0.2613 (0.1667 for retained outcome significance) and a partial *R*^2^ of 0.0846 for the treatment-outcome relationship.

### 3.5 Exploratory *post-hoc* analysis

Results of the exploratory *post-hoc* analysis are shown in Appendix Tables A1–A3 and Figure A1. ARR, hazard of relapse, MRI activity, and NEDA-3 loss were lower in patients on antibody-based DMTs than in those on S1P receptor modulators or cladribine, while CDP and treatment discontinuation hazards did not differ.

## 4 Discussion

In this PS-matched cohort study, we assessed the real-world effectiveness of HE vs. LE DMTs in both treatment-naïve and previously treated patients at the group level, acknowledging that treatment decisions in practice are individualized and no single DMT is universally preferred. As a main result, HE DMTs were more effective than LE DMTs in preventing relapses, inhibiting MRI activity, and maintaining NEDA-3 status. In addition, treatment discontinuation was significantly less frequent in the HE DMT group.

Efficacy estimates of DMTs and their classification into HE and LE DMTs are largely based on pivotal Phase III trials. Head-to-head trials have shown that alemtuzumab, ocrelizumab, ofatumumab, ublituximab, ozanimod, and ponesimod result in significantly lower ARR than interferons or teriflunomide, with relative reductions ranging from 31% for ponesimod (35) to 58% for ofatumumab (36) and 59% for ublituximab (37). Phase III placebo-controlled trials of fingolimod, cladribine, and natalizumab reported relative ARR reductions between 54% for fingolimod (38) and 68% for natalizumab (4). Importantly, real-world studies focusing on treatment-naïve patients have reported comparable group-level ARR reductions of 50%−60% when comparing HE vs. LE DMTs (14, 15).

While the ARR reduction observed in our study is broadly consistent with findings from Phase III trials and other real-world cohorts, the 64% relative reduction between HE and LE DMTs slightly surpasses the efficacy estimates reported in head-to-head comparisons. This difference is primarily driven by the higher relapse rate in the LE DMT group (ARR 0.37) compared to most Phase III LE comparator arms (35–37, 39, 40), rather than an unusually low ARR in the HE DMT group (ARR 0.13). Sensitivity analyses ruled out group differences in follow-up time and visit intervals as explanations. A likely factor contributing to a more pronounced relative ARR reduction is the higher disease activity in our cohort, reflected by a 47%−49% prevalence of gadolinium-enhancing (Gd+) MRI lesions at baseline, which exceeds rates in most pivotal trials by 5–10 percentage points (35–37, 39, 41). In HE DMT trials, including cladribine (42), ocrelizumab (43) and ublituximab (44), subgroup analyses have shown greater ARR reduction in patients with higher disease activity, including those with Gd+ MRI lesions, suggesting that HE DMTs suppress inflammation more robustly than LE DMTs. For instance, in the ULTIMATE trials, patients with Gd+ MRI lesions at baseline experienced a stronger treatment effect, with an ARR reduction of ~66% under ublituximab, compared to ~41% in Gd– patients (44).

While ARR is the typical primary endpoint in Phase III RCTs, several trials have also assessed NEDA-3, a composite of relapses, CDP, and MRI activity (23), as a secondary or exploratory outcome. NEDA-3 has previously been proposed as a key treatment goal in RRMS (45). In our study, HE DMTs were associated with a 53% lower hazard of losing NEDA-3, suggesting sustained superiority in controlling clinical and radiological disease activity. This aligns with Phase III head-to-head trials, where B-cell depleting therapies (ofatumumab, ocrelizumab, ublituximab) and S1P receptor modulators (ponesimod, ozanimod) were significantly superior in achieving NEDA-3 status than teriflunomide (35, 37, 46) or interferon β-1a (47, 48). However, direct cross-trial comparisons of NEDA-3 status remain challenging due to variations in NEDA-3 definitions, outcome components, study length and different metrics (45). In our study, the lower hazard of losing NEDA-3 with HE DMTs was largely driven by MRI activity, reflected in a 63% lower hazard of new or enlarging T2 lesions—a pattern consistent with HE Phase III trials. Reported reductions range from 48% [ozanimod vs. interferon β-1a (40)] to 85% [ofatumumab vs. teriflunomide (36)].

In our study, the hazard of CDP did not differ significantly between groups, though HE DMTs showed a numerical advantage. This suggests their primary benefit lies in suppressing acute inflammatory activity, as reflected by the significantly lower ARR, rather than mitigating neurodegeneration-driven disability progression. Similar findings have been reported in HE Phase III trials (49) and real-world cohorts (50), indicating that while HE DMTs effectively suppress relapse-associated worsening, their impact on progression independent of relapse activity may be more limited. Importantly, the absence of a statistically significant difference in CDP should be interpreted in light of the limited number of progression events, the relatively short follow-up for disability outcomes, and the variability inherent to EDSS assessments performed in routine clinical practice (51).

HE DMTs are associated with higher safety concerns, particularly infection risks (11–13). For example, a nationwide cohort study reported rituximab had the highest rate of serious infections among HE and LE DMTs (52). However, in our study, the hazard of treatment discontinuation was 67%−87% lower in the HE DMT group, primarily driven by breakthrough disease in the LE DMT group. This suggests that safety concerns do not necessarily translate into reduced treatment persistence within the observed follow-up period. Similar patterns have also been reported in other real-world cohorts (14, 53). For example, compared to rituximab, the hazard of treatment discontinuation per year was substantially higher with interferon beta, glatiramer acetate, or dimethyl fumarate, with disease breakthrough as the main reason for discontinuation (53).

While many authors classify HE DMTs as we did, a definitive classification remains lacking (11). Even though S1P receptor modulators have demonstrated superior efficacy over LE DMTs in Phase III trials (35, 40), some guidelines consider S1P receptor modulators and cladribine as having intermediate effectiveness (9, 54). Indeed, our exploratory analysis suggests that antibody-based DMTs may be more effective than these oral therapies, aligning with real-world data (55) and network meta-analyses (56). However, differences within the HE group may have limited clinical relevance, as not all patients are eligible for antibody-based therapies. Moreover, oral HE DMTs may offer advantages beyond efficacy. Cladribine, for example, combines high efficacy with a low treatment burden and temporary immunosuppression, as highlighted in a recent Cochrane review (57).

### 4.1 Limitations

To reduce confounding by indication and baseline imbalances inherent to non-randomized designs, we applied PS matching using a comprehensive set of prespecified covariates known to predict outcomes. A particular strength of our study is the availability of baseline MRI data for all patients, enabling a detailed assessment of disease activity at study entry.

PS matching resulted in well-balanced groups, as indicated by most SMDs < 0.1. Although minor residual differences remained, including a higher proportion of previously HE-treated patient, the corresponding SMD value of 0.19 can still be considered acceptable (28). To further address attrition and detection bias, we performed sensitivity analyses using pairwise censoring and adjustments for visit and MRI frequency (58), confirming the robustness of our results. Unlike RCTs, our study reflects real-world treatment patterns in patients with RRMS. However, the observational study design and reliance on routinely collected clinical data are associated with inherent limitations. The exclusion of observations with incomplete baseline data may have introduced a degree of selection bias. Furthermore, factors potentially influencing treatment decisions and outcomes - such as patient preferences, comorbidities, and physician recommendations - may not be fully recorded. As residual confounding due to unmeasured variables including effects related to treatment selection and sequencing cannot be ruled out, we performed another sensitivity analysis to quantify the strength of confounding that would be required to nullify the observed effects, yielding robustness values that may inform future study comparisons (30). As treatment episodes with < 6 months of therapy were excluded by design to allow observation of therapeutic effects (19), very early treatment discontinuations - most likely related to tolerability - were not captured. Because LE DMTs may be subject to earlier switching thresholds in routine care, often triggered by relapse, and follow-up was censored at treatment switch, relapse events may have contributed to shorter exposure times, which may result in upwardly biased ARR estimates for this group. As a single-center study, the generalizability of our findings may be limited. Nevertheless, the inclusion of both treatment-naïve and previously treated patients, along with the extended follow-up period, supports the external validity of the results.

## 5 Conclusion

In this real-world, PS-matched cohort study, HE DMTs grouped according to current classifications were associated with significantly lower ARR and reduced hazards of MRI activity and NEDA-3 loss. Moreover, treatment persistence was higher in the HE DMT group under real-world conditions. These findings are consistent with Phase III trial data and support the early and broad implementation of HE DMTs in MS management. LE DMTs may remain appropriate in selected clinical situations, such as during pregnancy or when HE DMTs are contraindicated.

## Data Availability

The raw data supporting the conclusions of this article will be made available by the authors, without undue reservation.
