# Supplementary material for: Real-world effectiveness of high-efficacy therapies in multiple sclerosis: a propensity score-matched cohort study
Source: Front Neurol. 2026 Mar 13;17:1773074. doi: 10.3389/fneur.2026.1773074 (PMC13021428; doi:10.3389/fneur.2026.1773074)
Supplement: Supplementary file 1 [file Table_1.DOCX]

**Appendix**

# **Table A1:** Cohort characteristics of an exploratory post-hoc analysis within the HE group comparing cladribine and S1P receptor modulators with antibody-based DMTs

| Characteristic | Unmatched cohort (n = 238) | | | PS matched cohort (n = 152) | | |
| --- | --- | --- | --- | --- | --- | --- |
|  | **Clad/S1P** (n = 86) | **AB-based** (n = 152) | **SMD** | **Clad/S1P** (n = 76) | **AB-based** (n = 76) | **SMD** |
| Age (years), median (IQR) | 38 (31-43) | 35 (29-43) | 0.18 | 37 (30-43) | 35 (30-43) | 0.10 |
| Female sex, n (%) | 60 (70%) | 113 (74%) | 0.10 | 52 (68%) | 53 (70%) | 0.03 |
| Time since symptom onset (years), median (IQR) | 7 (2-11) | 7 (2-12) | 0.04 | 6.8 (2.4-11.4) | 5.9 (1.7-10.5) | 0.07 |
| Time since MS diagnosis (years), median (IQR) | 4.8 (1.6-9.1) | 5.7 (1.1-10.4) | 0.10 | 4.5 (1.5-8.9) | 3.7 (0.9-9.2) | 0.01 |
| EDSS score, median (IQR) | 2.3 (1.5-3.5) | 2.0 (1.5-3.5) | 0.09 | 2.0 (1.5-3.5) | 2.0 (1.4-3.0) | 0.08 |
| Relapses last year, median n (IQR) | 1.0 (0.3-1.0) | 1.0 (1.0-2.0) | 0.30 | 1.0 (1.0-2.0) | 1.0 (0.8-1.3) | 0.00 |
| MRI T2 lesions, n (%) |  |  | 0.20 |  |  | 0.04 |
| 1-2 | 0 (0%) | 2 (1.3%) |  | 0 (0%) | 0 (0%) |  |
| 3-8 | 10 (12%) | 23 (15%) |  | 10 (13%) | 9 (12%) |  |
| ≥9 | 76 (88%) | 127 (84%) |  | 66 (87%) | 67 (88%) |  |
| Contrast enhancing MRI lesions, n (%) | 32 (37%) | 76 (50%) | 0.26 | 31 (41%) | 36 (47%) | 0.13 |
| CDP last year, n (%) | 20 (23%) | 40 (26%) | 0.07 | 19 (25%) | 19 (25%) | 0.00 |
| Previous DMT, median n (IQR) | 1 (1-2) | 2 (1-2) | 0.20 | 1 (1-2) | 2 (1-2) | 0.07 |
| Most effective previous DMT, n (%) |  |  | 0.44 |  |  | 0.08 |
| None | 15 (17%) | 27 (18%) |  | 14 (18%) | 15 (20%) |  |
| LE DMT | 48 (56%) | 55 (36%) |  | 39 (51%) | 36 (47%) |  |
| HE DMT | 23 (27%) | 70 (46%) |  | 23 (30%) | 25 (33%) |  |
| Follow-up time (months), median (IQR) | 43 (26-62) | 43 (20-70) | 0.02 | 44 (26-61) | 40 (18-66) | 0.16 |
| Visit intervals (months), median (IQR) | 5 (4-7) | 3 (3-4) | 1.0 | 5 (3-6) | 3 (3-4) | 0.93 |
| MRI intervals (months), median (IQR) | 13 (10-16) | 12 (9-12) | 0.42 | 13 (10-15) | 12 (9-12) | 0.42 |
| Actual DMT, n (%) |  |  |  |  |  |  |
| Fingolimod | 47 (55%) | - |  | 43 (57%) | - |  |
| Cladribine | 35 (41%) | - |  | 29 (38%) | - |  |
| Ozanimod | 4 (4.7%) | - |  | 4 (5.3%) | - |  |
| Natalizumab | - | 62 (41%) |  | - | 29 (38%) |  |
| Alemtuzumab | - | 26 (17%) |  | - | 12 (16%) |  |
| Ocrelizumab | - | 48 (32%) |  | - | 23 (30%) |  |
| Ofatumumab | - | 16 (11%) |  | - | 12 (16%) |  |

EDSS: Expanded Disability Status Scale; CDP: 3-month confirmed disability progression; DMT: disease-modifying therapy; LE: low-efficacy; HE: high-efficacy; MRI: magnetic resonance imaging; n: number of observations; PS: propensity score; IQR: interquartile range; SMD: absolute standardized mean difference; Clad: Cladribine; S1P-receptor modulators: Fingolimod, Ozanimod; AB-based: Antibody-based DMT (Alemtuzumab, Ocrelizumab, Ofatumumab, Natalizumab). Covariates used for PS estimation are shown in bold. An SMD of ≤ 0.1 defines adequate balance between the treatment groups.

**Table A2:** ARR of an exploratory post-hoc analysis within the HE group comparing cladribine and S1P receptor modulators with antibody-based DMTs

| **DMT** | **ARR** | **95%-CI** | **RR** |
| --- | --- | --- | --- |
| Cladribine / S1P-receptor modulator | **0.26** | 0.20 – 0.35 | **0.31**  (95% CI: 0.16 – 0.56; p = 0.0003) |
| Antibody-based DMT | **0.08** | 0.05 – 0.14 |  |

ARR: annualized relapse rate; CI: confidence interval; RR: rate ratio; DMT: disease-modifying therapy. S1P-receptor modulators: Fingolimod, Ozanimod; Antibodies: Alemtuzumab, Ocrelizumab, Ofatumumab, Natalizumab. P-value results from the wald test.

**Table A3:** Secondary endpoints of an exploratory post-hoc analysis within the HE group comparing cladribine and S1P receptor modulators with antibody-based DMTs

| Endpoints | Hazard ratio | 95 % CI | p-value |
| --- | --- | --- | --- |
| Relapse | 0.27 | 0.15 - 0.51 | <0.001 |
| MRI activity | 0.45 | 0.26 - 0.78 | 0.004 |
| CDP | 0.76 | 0.37 - 1.55 | 0.4 |
| Loss of NEDA-3 | 0.41 | 0.26 - 0.64 | <0.001 |
| Treatment discontinuation | 0.92 | 0.54 - 1.59 | 0.8 |

CDP: 3-month confirmed disability progression; CI: confidence interval; DMT: disease-modifying therapy; HE: high-efficacy; MRI: magnetic resonance imaging; NEDA: no evidence of disease activity. P-values result from the likelihood-ratio test. Hazard ratio < 1 favors antibody-based DMTs.

**Figure A1:** Kaplan–Meier estimates of secondary outcomes of an exploratory post-hoc analysis within the HE group comparing cladribine and S1P receptor modulators with antibody-based DMTs


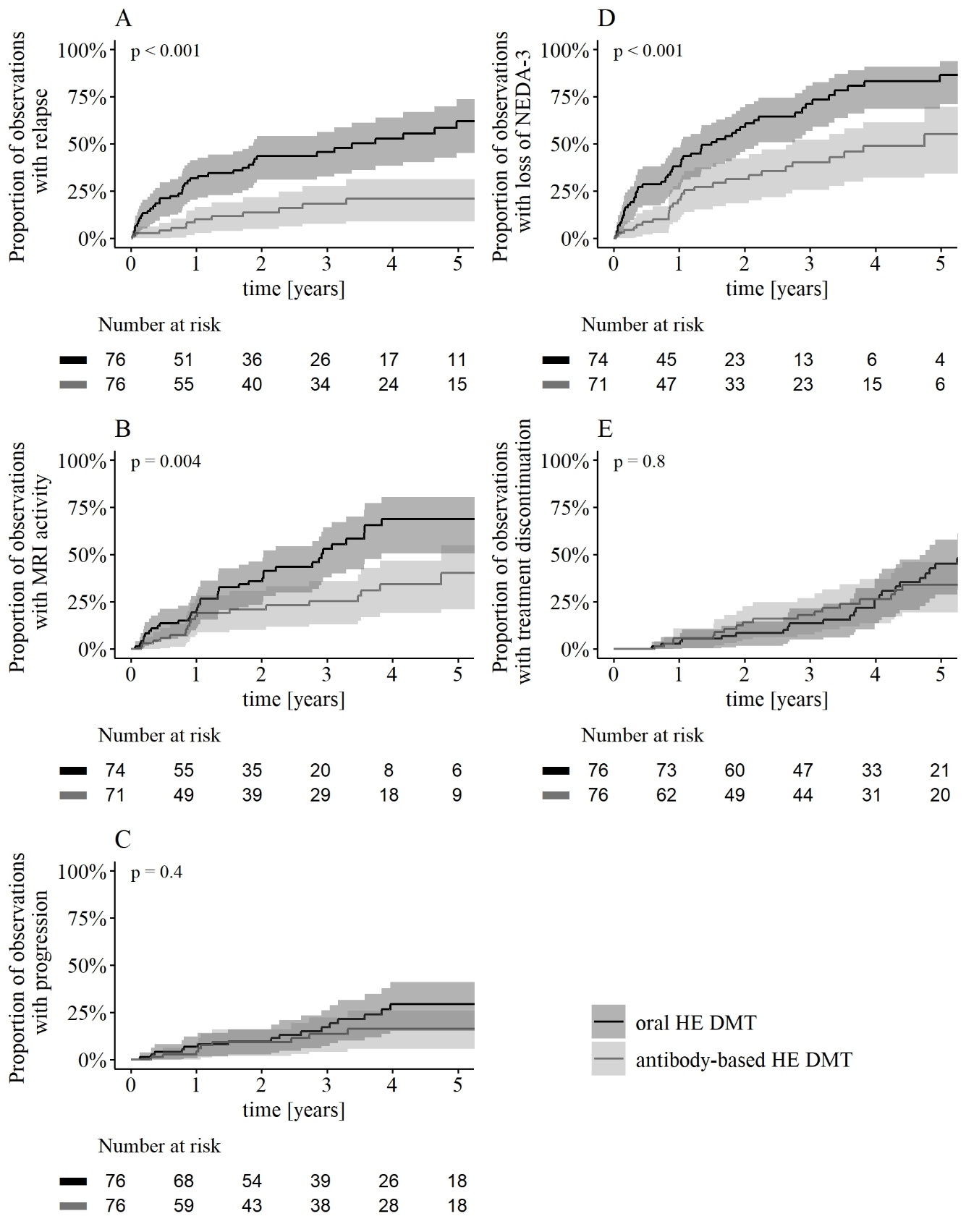


Kaplan–Meier estimates and log-transformed pointwise 95% confidence intervals showing the proportion of observations experiencing the respective event over time. P-values result from log-rank test. Observations without follow-up MRI were excluded for MRI-based endpoints (B, D; oral HE DMT n=2, antibody-based HE DMT n=5). Patients who discontinued treatment within the first 6 months were excluded by design, as reflected in the Kaplan-Meier plot by a flat line during this period (E). Antibody-based HE-DMT: Alemtuzumab, Ocrelizumab, Ofatumumab, Natalizumab; oral HE-DMT: Cladribine, S1P-receptor modulators. DMT: disease-modifying therapy; HE: high-efficacy; MRI: magnetic resonance imaging; CDP: 3-month confirmed disability progression; NEDA: no evidence of disease activity.
